# Phase Transformation during the Selective Dissolution of a Cu_85_Pd_15_ Alloy: Nucleation Kinetics and Contribution to Electrocatalytic Activity

**DOI:** 10.3390/ma16041606

**Published:** 2023-02-15

**Authors:** Frol Vdovenkov, Eugenia Bedova, Oleg Kozaderov

**Affiliations:** Department of Physical Chemistry, Faculty of Chemistry, Voronezh State University, Voronezh 394018, Russia

**Keywords:** alloy, copper, palladium, selective anodic dissolution, phase transformation, heterogeneous nucleation, kinetics, formic acid, electro-oxidation, electrocatalytic activity

## Abstract

This study determined the critical parameters for the morphological development of the electrode surface (the critical potential and the critical charge) during anodic selective dissolution of a Cu–Pd alloy with a volume concentration of 15 at.% palladium. When the critical values were exceeded, a phase transition occurred with the formation of palladium’s own phase. Chronoamperometry aided in the determination of the partial rates of copper ionization and phase transformation of palladium under overcritical selective dissolution conditions. The study determined that the formation of a new palladium phase is controlled by a surface diffusion of the ad-atom to the growing three-dimensional nucleus under instantaneous activation of the nucleation centres. We also identified the role of this process in the formation of the electrocatalytic activity of the anodically modified alloy during electro-oxidation of formic acid. This study demonstrated that HCOOH is only oxidated at a relatively high rate on the surface of the Cu_85_Pd_15_ alloy, which is subjected to selective dissolution under overcritical conditions. This can be explained by the fact that during selective dissolution of the alloy, a pure palladium phase is formed on its highly developed surface which has prominent catalytic activity towards the electro-oxidation of formic acid. The rate of electro-oxidation of HCOOH on the surface of the anodically modified alloy increased with the growth of the potential and the charge of selective dissolution, which can be used to obtain an electrode palladium electrocatalyst with a set level of electrocatalytic activity towards the anodic oxidation of formic acid.

## 1. Introduction

Selective dissolution of alloys is a promising way to obtain new electrocatalytic materials with highly developed surfaces and increased activity that can be used in energy conversion devices [1]. The process involves corrosive or anodic dissolution of the electronegative component, with the electropositive component remaining electrochemically stable [2,3].

The synthesis of palladium electrocatalysts by means of selective dissolution used for anodic oxidation of hydrogen or organic compounds, as well as cathodic oxygen reduction, is of great interest when used for the design of fuel cells, hydrogen accumulators and electrochemical sensors [4,5,6]. A highly porous and stable electrocatalyst with relatively high specific activity of hydrogen evolution reaction was designed in [7,8] by means of selective electrochemical dissolution of Zr from quasi-crystalline PdZr_3_ metallic glasses and nickel from Pd–Ni films. The dealuminified PdCuAl alloys and palladium deposited on the dealloyed Au_50_Ag_50_ nanoporous structure proved to be very effective in catalysing oxygen reduction [9,10].

The use of the highly developed palladium material obtained by selective dissolution is of particular interest in reactions involving organic compounds. For instance, Pd materials with a uniform bicontinuous and interpenetrating three-dimensional structure prepared from palladium-containing metallic glasses [11,12] and alloys [13,14,15,16] exhibited enhanced catalytic activity towards formic acid and methanol electro-oxidation.

Anodic selective dissolution is more preferable than selective corrosion to engineer the surface structure of palladium catalysts for electrocatalytic reactions, since it allows control of the phase composition and the morphology of the final product by changing the electrode potential and the imposed electric charge. It is crucially important to determine the dependence between the kinetics of selective dissolution of an alloy and the electrocatalytic properties of the formed material. By doing this, we can determine the optimal conditions for the synthesis of an electrocatalyst with a determined surface activity.

In our study, we analysed a Cu–Pd alloy containing 15 at.% palladium. This alloy selectively dissolves in an acidic sulphate solution at anodic polarization [17]. The electrochemical behaviour of selectively dissolved alloys with a prevailing electronegative component is characterised by the rapid acceleration of the anodic process when a critical potential *E*_cr_ is exceeded [3,18,19]. This potential corresponds to the initiation of significant morphological changes on the surface of the alloy, whose roughness factor increases dramatically, including for Cu–Pd alloys [20]. With subcritical potentials *E* < *E*_cr_, diffusion-controlled selective dissolution of the atoms of the electronegative component results in an increase in the concentration of nonequilibrium defects in the surface layer of the alloy [21]. As demonstrated in [17], the structural and vacancy defectiveness of the Cu–Pd alloy increased as a result of subcritical selective dissolution as the key factor leading to an increase in its electrocatalytic activity observed during the anodic oxidation of oxalic acid.

However, the effect is significantly greater when the alloy is selectively dissolved at overcritical potentials *E* > *E*_cr_. Such conditions make it thermodynamically possible for the formation of the palladium phase with a highly developed morphology and increased surface energy [22,23]. An essential condition for such a phase transformation is the dissolution of a certain amount of copper, which results in the supersaturation of the surface layer with nonequilibrium defects, for instance, vacancies (□). In other words, some electric charge *q*_cr_ should go through the electrode corresponding to a certain critical concentration of defects. If the electrochemical parameters of selective dissolution are *E* >> *E*_cr_ and *q* >> *q*_cr_, the system becomes absolutely unstable, and the phase transformation can be described in terms of activationless spinodal decomposition [24,25]. However, if the potential and the charge exceed the critical values only slightly and the components of the alloy are characterised by a sufficiently low diffusion mobility (as in case of Cu–Pd alloy), the energy barrier of the phase transformation is significant and its kinetics can be described in terms of irreversible heterogeneous nucleation and the growth of a new phase [26,27,28].

It is obvious that in order to obtain a material with a certain electrocatalytic activity, it is necessary to determine the connection between the main parameters of the phase transformation during selective dissolution (electrode potential, imposed charge) and the rate of the electrocatalytic process on the surface of the anodically modified alloy. In our study, we determined this dependence based on selective dissolution of the Cu–Pd alloy and anodic oxidation of formic acid, which is a promising reducing agent for fuel cells based on solutions of organic compounds.

The purpose of our study was to determine the kinetic regularities of palladium nucleation during selective dissolution of a copper-based Cu–Pd alloy. We also analysed the role of this process in the formation of the electrocatalytic activity of the anodically modified alloy during the electro-oxidation of formic acid.

## 2. Materials and Methods

In our study, we used a polycrystalline Cu–Pd alloy with an atomic ratio of palladium *x*_Pd_ = 0.15. The alloy was prepared by direct smelting in a tungsten induction furnace preliminarily evacuated and filled with argon (1.2 atm) in Al_2_O_3_ crucibles. The metals were kept in a molten state at 1723 K, then cooled to 1373 K at a speed of 600 K/h, after which they were quench hardened in water. According to the phase diagram and quench hardening mode, the obtained alloy was a statistically disordered solid solution.

In order to make the electrode, the alloy was cut, polished and placed in a frame of polymerized epoxy resin. Standard preparation of the electrode surface included striping on sanding paper with decreasing grain size, polishing on chamois with an aqueous MgO suspension with grains of an average size of ~50 μm to a mirror finish, washing with distilled water, degreasing with ethyl alcohol, followed by washing with bidistilled water and drying with filter paper.

The anodic modification of the alloy involved its selective dissolution. It was conducted in a 5 mM CuSO_4_ + 0.5 mM H_2_SO_4_ + 0.05 M Na_2_SO_4_ solution prepared in bidistilled water from chemically pure salts and extra pure concentrated sulphuric acid. Addition of CuSO_4_ to the electrolyte contributed to a faster establishment and stabilization of the open-circuit potential. The electrocatalytic activity of the Cu–Pd alloy subjected to selective dissolution was studied during the electro-oxidation of formic acid in a 1 M HCOOH + 0.05 M H_2_SO_4_ solution. To remove the dissolved oxygen prior to the electrochemical measurements, the working solutions were deaerated with chemically pure argon directly in the cell for at least 2 h. The experiments were conducted in non-mixed solutions.

In our study, we used a standard three-electrode cell without separation of the spaces of the working and auxiliary electrodes. A platinum plate (geometric surface area = 1.6 cm^2^) was used as an auxiliary electrode. The saturated silver/silver chloride reference electrode was located in a separate vessel and connected to the cell by an electrolytic bridge filled with a saturated solution of ammonium nitrate. The reference electrode potential referring to a standard hydrogen electrode was *E*_ref_ = 201 mV. The potentials *E* in the study are presented relative to the standard hydrogen electrode scale, and were calculated by adding *E*_ref_ to the measured value of the working electrode potential.

The change and maintenance of the electrode potential, as well as the registration of polarization curves, were performed using an IPC-Pro-L (Volta, Saint-Petersburg, Russia) computerized potentiostat unit. The electrode prepared for the experiment was placed in a cell filled with a deaerated working solution and incubated until the quasi-stationary value of the open-circuit potential was established. For potentiodynamic measurements, the potential scan rate was set at *v* = d*E*/d*t* and the polarization of the *i*,*E* curve was recorded. During the potentiostatic measurements, the potential *E* = const was set and *i*,*t*-curve (chronoamperogram) was recorded for some time. Selective dissolution of the alloys was performed at different anodic potentials *E*_mod_ and imposed electric charges *q*_mod_.

**Figure 1:**
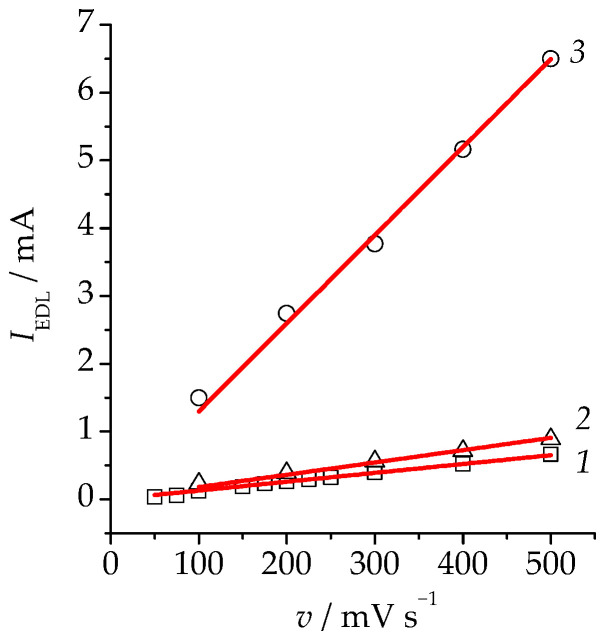
Dependence of the charging current of the EDL at *E* = 50 mV on the potential scan rate, obtained in 0.5 M H_2_SO_4_ for (1)—unmodified alloy, (2)—anodically modified Cu_85_Pd_15_ (*E*_mod_ = 630 mV, *q*_mod_ = 24 mC·cm^−2^) alloy, and (3)—anodically modified Cu_85_Pd_15_ (*E*_mod_ = 630 mV, *q*_mod_ = 480 mC·cm^−2^) alloy.

Current density *i* was calculated by normalizing the current strength per unit (visible) geometric area of the electrode. To determine the development of the electrode surface during selective dissolution of the alloy, the relative roughness factor was calculated as the ratio of the integral electric double-layer (EDL) capacitances. For this, the studied electrode was put in a 0.5 M H_2_SO_4_ solution. Then, in a continuous mode, we obtained 20 cyclic *i*,*E* curves in the range of potentials in the charging region of the EDL (from −150 mV to 50 mV) with a potential scan rate from 25 to 500 mV/s. Having determined the charging current *I*_EDL_, we plotted its dependence on the potential scan rate (Figure 1). Taking into account the fact that the slope of the line corresponds to the integral double-layer capacitance, for the ratio of the slopes of the modified and the initial alloys the relative roughness factor *f*_r_, i.e., the degree of the development of the surface of the alloy, was calculated.

## 3. Results

**Figure 2:**
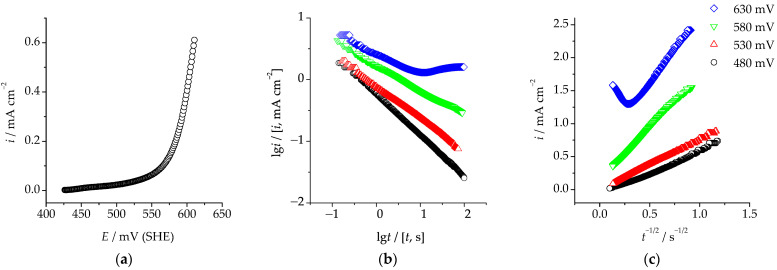
(**a**)—Anodic polarization curve, (**b**)—anodic chronoamperograms in double logarithmic coordinates, (**c**)—anodic chronoamperograms in the Cottrell coordinates obtained on the Cu_85_Pd_15_ alloy in 5 mM CuSO_4_ + 0.5 mM H_2_SO_4_ + 0.05 M Na_2_SO_4_ at the potential scan rate of 1 mV/s.

The anodic polarization curve obtained in an acidified sulphate solution on the Cu_85_Pd_15_ solution at the potential scan rate of 1 mV/s is presented in Figure 2a. It is obvious that the polarization curve can be divided in two parts. In the low-current region, the rate of the anodic process is low and weakly depends on the potential. The corresponding current density is proportional to the rate of selective dissolution of the electronegative component. When a certain critical potential *E*_cr_ was exceeded, a sharp increase in the anodic current density was observed. The critical potential determined for the Cu_85_Pd_15_ alloy by means of extrapolation of the high-current region on the potential axis was *E*_cr_ = 575 ± 8 mV.

To determine the kinetic regularity of the regrouping of palladium into its own phase at overcritical potentials, we obtained chronoamperograms of the selective dissolution of the studied alloys in the vicinity of *E*_cr_ (Figure 2b,c). We can see that irrespective of the anodic potential, the rate of anodic selective dissolution of the Cu–Pd alloy decreased over time, i.e., the process was nonstationary. At the same time, while being overall nonlinear, potentiostatic curves corresponding to the current decay became partially linear both in the double logarithmic and the Cottrel coordinates. However, the linearization was only observed at time moments below a certain critical value *t*_cr_, which corresponds to the beginning of phase transformations in the surface layer of the alloy supersaturated with nonequilibrium vacancies [22,23]. Moment *t*_cr_ corresponds to the flow of the critical charge $q_{\mathrm{cr}}=\int_{0}^{t_{\mathrm{cr}}}i\left(t\right)dt$ determined for the alloy through the electrode. Integration of the chronoamperograms of selective dissolution obtained at the overcritical potential *E*_mod_ = 630 mV in the range from 0 to *t*_cr_ results in *q*_cr_ ≈ 13 mC·cm^−2^. 

When *t* < *t*_cr_, only one process can take place in the system, namely the ionization of copper. Therefore, the observed linearization of a part of the bilogarithmic curve corresponding to the current decay with a slope of ~0.5, might indicate the diffusion character of kinetic limitations of the process under subcritical polarization conditions, i.e., when *t* < *t*_cr_ and *q* < *q*_cr_. This is confirmed by partial linearization of the chronoamperograms in the Cottrell coordinates (Figure 2c) at relatively small polarization times.

Based on the chronoamperometry data and using the approach developed in [29] for kinetic description of the phase transformation of the electropositive component, when *E*_cr_ and *q*_cr_ are comparatively slightly exceeded, we can suggest the following scheme of the anodic process for the Cu–Pd alloy:

(1) $$\;\;\;\left[\begin{array}{c} \mathrm{Cu}\\ |\\ \mathrm{Pd} \end{array}\right]\begin{array}{l} \overset{\mathrm{mass}\text{-}\mathrm{transfer}}{\underset{\mathrm{in}\;\mathrm{alloy}}{\to }}Cu_{\left(s\right)}\overset{\mathrm{ionization}}{⇄}\;□\;+\;2e+Cu_{\left(s\right)}^{2+}\overset{\mathrm{mass}\text{-}\mathrm{transfer}}{\underset{\mathrm{in}\;\mathrm{solution}}{\to }}Cu_{\left(v\right)}^{2+}\\ \overset{\mathrm{mass}\text{-}\mathrm{transfer}}{\underset{\mathrm{in}\;\mathrm{alloy}}{\to }}Pd_{\left(s\right)}+\;□\;\overset{\mathrm{activation}}{\underset{\mathrm{deactivation}}{⇄}}Pd_{\left(s\right)}^{*}\overset{\mathrm{nucleation}}{\underset{\mathrm{decomposition}}{⇄}}Pd_{3D} \end{array}$$

We can see that in the general case the process involves solid state and liquid state mass transfer stages, as well as the phase transition of palladium from the nonequilibrium state Pd* to a new phase Pd_3D_. Assuming that the formation and growth of the new palladium phase proceeds according to the heterogeneous nucleation process, taking into account the fact that phase transformation starts when *t* > *t*_cr_ and following the model developed in [29], we obtain the following expression for the general current density registered in the circuit:

(2) $$i_{\mathrm{Cu}}\left(t\right)=\left\{\begin{array}{l} i_{\mathrm{Cu}}^{\mathrm{mass}}\left(t\right)\;\;\mathrm{at}\;\;t\leq t_{\mathrm{cr}};\\ i_{\mathrm{Cu}}^{\mathrm{mass}}\left(t\right)+i_{\mathrm{Pd}}^{\mathrm{nucl}}\left(t\right)\;\;\mathrm{at}\;\;t>t_{\mathrm{cr}}. \end{array}\right.$$

Here, $i_{\mathrm{Pd}}^{\mathrm{nucl}}\left(t\right)$ is the additional contribution to the current of copper ionization caused by the phase regrouping of palladium, which results in the opening of the underlying layers of the alloy and their contact with the electrolyte. We should note that $i_{\mathrm{Pd}}^{\mathrm{nucl}}\left(t\right)$ is not connected with the partial Faraday current of the electrode reaction with Pd. It is proportional to the flux density of its phase regrouping.

The algorithm for the calculation of the partial current density $i_{\mathrm{Pd}}^{\mathrm{nucl}}\left(t\right)$ based on the experimental data suggested in [30], takes into account the fact that, in a general case, the current density of Cu dissolution caused by mass transfer can be presented as follows:

(3) $$i_{\mathrm{Cu}}^{\mathrm{mass}}\left(t\right)=\mathrm{const}\cdot t^{-m}$$

Having determined m and const based on the linear region of the experimental chronoamperogram replotted in the double logarithmic coordinates, we can calculate $i_{\mathrm{Cu}}^{\mathrm{mass}}\left(t\right)$ when *t* > *t*_cr_. Using the difference between the overall registered current in the circuit $i\left(t\right)$ and the calculated density of the diffusion current $i_{\mathrm{Cu}}^{\mathrm{mass}}\left(t\right)$ we calculated the current density transient corresponding to the phase formation process involving palladium:

(4) $$i_{\mathrm{Pd}}^{\mathrm{nucl}}\left(t\right)=i\left(t\right)-\mathrm{const}\cdot t^{-m}$$

The kinetic analysis of the phase transition of palladium was conducted using 3D nucleation models. The limiting stage was the incorporation of palladium ad-atoms (kinetic mode) or its surface diffusion (diffusion mode) to the growing three-dimensional nucleus following the instantaneous or continuous activation of the nucleation centres [31]. The initial regions of the current transient of the phase regrouping of palladium were replotted in the corresponding coordinates (Table 1).

The kinetic dependences were also compared with theoretical curves of 3D nucleation, which describe the whole process of formation of a new phase from the appearance and growth of isolated nuclei to the growth of partially overlapping nuclei and the new phase [31].

**Figure 3:**
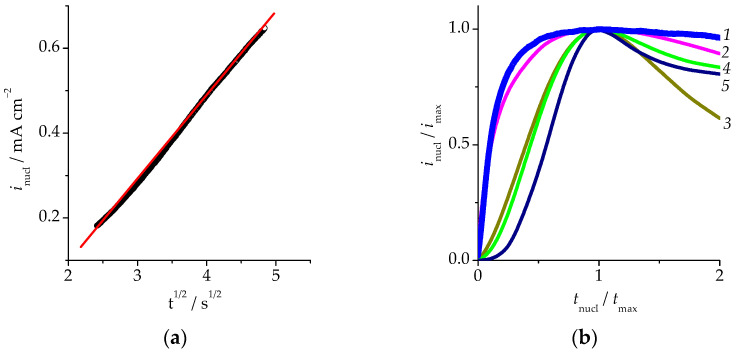
Current transient of palladium nucleation during overcritical selective dissolution of the Cu_85_Pd_15_ alloy at the overcritical potential *E*_mod_ = 630 mV: (**a**)—linearization of the initial region of the nucleation chronoamperogram in criteria coordinates for the mode of instantaneous activation of the nucleation centres under diffusion-controlled growth of the 3D nucleus: black points—experimental data; red line—linear regression; (**b**)—comparison of the experimental nucleation curve (1) with the theoretically calculated ones for various modes of 3D nucleation: (2)—instantaneous activation of the nucleation centres under diffusion-controlled growth of the 3D nuclei; (3)—continuous activation of the nucleation centres under diffusion-controlled growth of the 3D nuclei; (4)—instantaneous activation of the nucleation centres under kinetically controlled growth of the 3D nuclei; (5)—continuous activation of the nucleation centres under kinetically controlled growth of the 3D nuclei.

It appeared that the linearization of the initial region of the current dependency was observed only in the criteria coordinates of the 3D nucleation in the surface-diffusion mode with instantaneous activation of the nucleation centres (Figure 3a). This conclusion was confirmed by the comparison of experimental and theoretical current transients *i*_nucl_/*i*_max_ − *t*_nucl_/*t*_max_ (Figure 3b) obtained by normalizing the *i*,*t* curve to the current density of the maximum *i*_max_ and the time required to reach the maximum *t*_max_.

**Figure 4:**
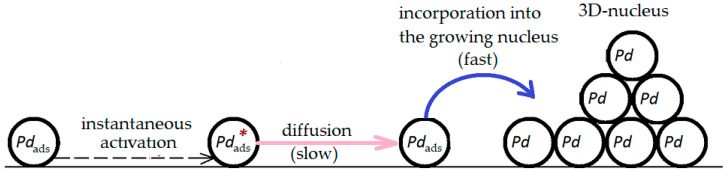
A scheme of the 3D nucleation of palladium during its phase transformation during anodic selective dissolution of the Cu–Pd alloy.

Thus, the overall process of anodic phase formation can be described with the stage-wise scheme presented in Figure 4.

**Figure 5:**
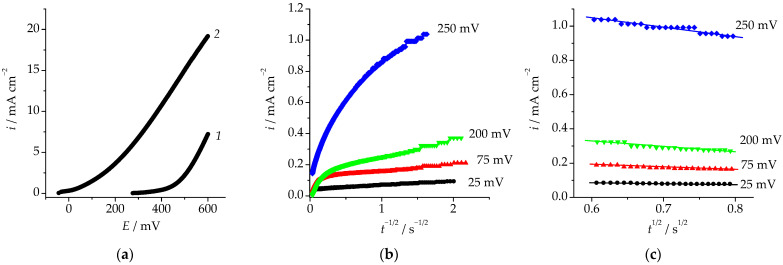
(**a**)—Polarization curves on the unmodified (1) and subjected to selective dissolution (2) Cu_85_Pd_15_ alloy in 1 M HCOOH + 0.05 M H_2_SO_4_; (**b**)—Cottrell chronoamperograms of the electro-oxidation of formic acid on the Cu_85_Pd_15_ alloy anodically modified at various potentials; (**c**)—initial regions of the electro-oxidation chronoamperograms of НСООН obtained from the Cu_85_Pd_15_ alloy anodically modified at various potentials and linearized in mixed-kinetic coordinates. Selective dissolution mode: *q*_mod_ = 24 mC·cm^−2^ and *E*_mod_ = 630 mV.

The effect of the phase transformations of palladium during selective dissolution of the Cu_85_Pd_15_ alloy on its electrocatalytic activity was studied based on the anodic oxidation of formic acid. An increase in the anodic current density during the transition from unmodified to the anodically modified alloy was observed when we compared electro-oxidation polarization curves (Figure 5a). We can also see that the open-circuit potential shifted towards negative values. This may be caused by a more effective chemisorption processes involving HCOOH on the alloy subjected to selective dissolution due to the formation of palladium’s own phase on its surface.

Electro-oxidation chronoamperograms of formic acid confirmed the nonstationary character of the process (Figure 5b). They were nonlinear in the Cottrell coordinates, while the current density of electro-oxidation of HCOOH increased with larger potentials. The shape of the *i*,*t* curves indicates the implementation of mixed-kinetic control when the diffusion mass transfer is complicated by a preliminary kinetic stage. In this case, at short time periods, the initial region of the chronoamperogram should be linearized in the coordinates *i* − *t*^1/2^ [32], which was indeed what we observed (Figure 5c). The y-intercept corresponds to the rate of the electrochemical stage *i*(0).

**Figure 6:**
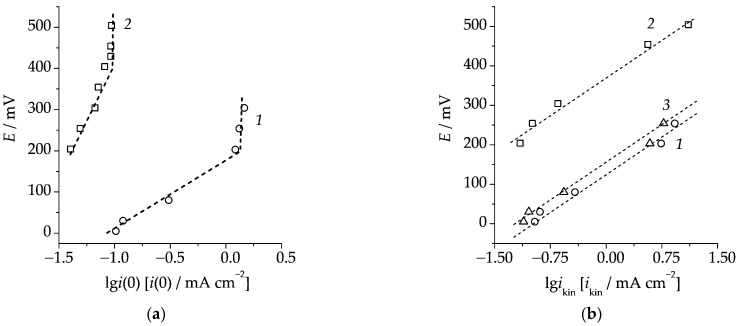
Tafel plots obtained in 1 M HCOOH + 0.05 M H_2_SO_4_ for (1)—Cu_85_Pd_15_ alloy after modification at *q*_mod_ = 24 mC·cm^−2^ and *E*_mod_ = 630 mV; (2)—palladium; (3)—Cu_85_Pd_15_ alloy after modification at *q*_mod_ = 24 mC·cm^−2^ and *E*_mod_ = 630 mV with correction for the degree of surface development. (**a**) without adjustment, and (**b**) with adjustment for the limiting current density of electro-oxidation.

Based on the kinetic currents *i*(0) determined for various anodic potentials of electro-oxidation, we obtained the semi-logarithmic Tafel plot, *E* vs. lg*i*(0), shown in Figure 6a together with a similar curve obtained for palladium.

As we assumed, *i*(0) increased with the growth of *E*. We should note that a significant acceleration of the kinetic stage of anodic oxidation of HCOOH was observed for the anodically modified alloy as compared with palladium. In both cases, *E*,lg*i*(0) curves were nonlinear within a wide range of potentials, and when the potentials were sufficiently large, the anodic current practically did not depend on *E*. We can assume that maximum current was reached when the chemical reaction preceding the electrochemical stage was hindered [32]. To determine the true rate of the electrochemical stage, the electro-oxidation current density was corrected considering the maximum current density using the formula:

(5) $$i_{\mathrm{kin}}=\frac{i\left(0\right)}{1-i\left(0\right)/i_{\lim}}$$

Here, *i*(0) is the current density of electro-oxidation at the initial moment; *i*_lim_ is the maximum current density of electro-oxidation; and *i*_kin_ is the current density corresponding to the kinetic stage of electro-oxidation of HCOOH.

The adjusted Tafel plots *E* vs. lg*i*_kin_ were linear (Figure 6b). Even considering the degree of surface development (curve 3), they had the same slope angle d*E*/dlg*i*_kin_ = 120 ± 8 mV corresponding to the single-electron charge transfer. Based on the obtained data, we can suggest the following possible scheme of the electro-oxidation of formic acid: the dissociative chemisorption stage of the HCOOH molecule is followed by electrochemical oxidation of the adsorbed hydrogen and the adsorbed HCOO_ads_ particle:

HCOOH → HCOOH_ads_,
HCOOH_ads_ → HCOO_ads_ + H_ads_,
HCOO_ads_ → CO_2_ ↑ + H^+^ + e^–^,
H_ads_ → H^+^ + e^–^.

The analysis demonstrated that the rate of electro-oxidation of formic acid on an anodically modified alloy at *E* = const is several times higher than that on palladium. Therefore, we can talk about the electrocatalytic effect of the anodically modified alloy on the electro-oxidation of formic acid. The effect can be explained by the formation of a catalytically active phase of palladium during phase transformation during overcritical selective dissolution of copper from the Cu–Pd alloy.

**Figure 7:**
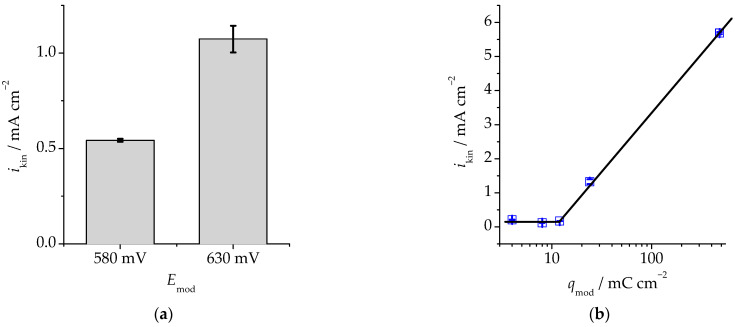
The effect of the potential (**a**) and the electric charge (**b**) of selective dissolution of the Cu_85_Pd_15_ alloy on the rate of the kinetic phase of electro-oxidation of formic acid. (**a**)—The imposed charge was constant, *q*_mod_ = 24 mC·cm^−2^; (**b**)—the anodic potential was constant, *E*_mod_ = 630 mV.

Next, we analysed the role of the main conditions of preliminary selective dissolution of the alloy on the rate of the kinetic stage of electro-oxidation of HCOOH. Thus, when transferring from the anodic modification at 580 mV to the anodic modification at 630 mV (in both cases the imposed charge was 24 mC·cm^−2^), *i*_kin_ doubled (Figure 7a). This is caused by the fact that the 630 mV potential is overcritical, which means that the anodic modification is accompanied by the phase transformation of palladium with the formation of its own highly developed phase on the surface of the electrode.

The imposed selective dissolution charge also significantly affected *i*_kin_ (Figure 7b), but only when a certain value was exceeded. This value apparently corresponds to the critical charge *q*_cr_ ≈ 13 mC·cm^−2^ determined based on the chronoamperometry data. Indeed, the current density of electro-oxidation on the alloy selectively dissolved at 2, 4 and 12 mC·cm^−2^ and 630 mV was low and did not depend on *q*_mod_. If the imposed charge was *q*_mod_ > 12 mC·cm^−2^, the kinetic current *i*_kin_ increased sharply (Figure 7b).

Thus, a significant increase in the rate of anodic oxidation of НСООН on the surface of the anodically modified Cu_85_Pd_15_ alloy was observed at overcritical potentials and overcritical electric charges of anodic selective dissolution.

**Figure 8:**
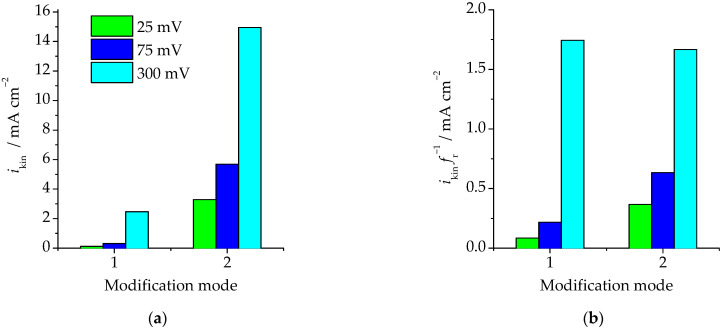
The rate of the kinetic stage of electro-oxidation of HCOOH on the anodically modified Cu_85_Pd_15_ alloy, found (**a**)—without, and (**b**)—with accounting for the surface development of the electrode depending on the potential of anodic oxidation of formic acid and the mode of anodic modification of the alloy: (1)—*E*_mod_ = 630 mV, *q*_mod_ = 24 mC·cm^−2^; (2)—*E*_mod_ = 630 mV, *q*_mod_ = 480 mC·cm^−2^.

We should bear in mind that this effect can be caused by either the growth of the electrocatalytic activity of the alloy, or the growth of its actual surface area. To differentiate between these factors, we calculated *i*_kin_ considering the degree of surface development of the electrode during its anodic modification (Figure 8).

Without taking into account the surface development, the current density of the electro-oxidation of HCOOH increased in the Pd < Cu_85_Pd_15_ (*E*_mod_ = 630 mV, *q*_mod_ = 24 mC·cm^−2^) < Cu_85_Pd_15_ (*E*_mod_ = 630 mV, *q*_mod_ = 480 mC·cm^−2^) sequence. The same tendency remained after the normalization of the current density to the degree of surface development of the alloy.

Therefore, selective dissolution of the Cu_85_Pd_15_ alloy at *E* > *E*_cr_ and *q* > *q*_cr_ only resulted in the formation of a surface layer which is electrocatalytically active towards anodic oxidation of НСООН. This can be explained by the fact that during anodic modification of the alloy, a pure palladium phase is formed on its surface which has prominent catalytic activity towards electro-oxidation of formic acid.

## 4. Conclusions

The critical potential for the surface development of the Cu_85_Pd_15_ alloy during its selective dissolution in an acidified sulphate aqueous solution was assessed using the polarization curve method. Chronoamperometry aided in the determination of the partial rates of the copper ionization and palladium phase transformation processes under overcritical polarization conditions. The study determined that the formation of a new palladium phase is controlled by a surface diffusion of the ad-atom to the growing three-dimensional nucleus under instantaneous activation of the nucleation centres. The rate increases with the anodic potential as compared to the potential critical for the start of the surface development.

Electro-oxidation of formic acid on the Cu_85_Pd_15_ alloy subjected to selective dissolution is likely to go through the dissociative chemisorption stage. This study determined that the rate of electro-oxidation of formic acid on the anodically modified alloy is several times higher than that on palladium, even taking into account the development of the electrode’s surface during selective dissolution of the alloy. This indicates the formation of electrocatalytic activity of the Cu_85_Pd_15_ alloy due to the formation of a new palladium phase on its highly developed surface. The electrocatalytic activity of the alloy can be regulated by altering the electrode potential and the electric charge of anodic selective dissolution which should exceed critical values corresponding to the beginning of phase and morphological transformations of the surface of the electrode.

## Figures and Tables

**Table 1:** Current transients of the anodic phase formation through 3D nucleation stages.

Activation Regime	Nucleus Growth Regime	Coordinates of Transient Linearization
Instantaneous	Diffusion	$i_{\mathrm{Pd}}^{\mathrm{nucl}}-t^{1/2}$
	Kinetic	$i_{\mathrm{Pd}}^{\mathrm{nucl}}-t^{2}$
Continuous	Diffusion	$i_{\mathrm{Pd}}^{\mathrm{nucl}}-t^{3/2}$
	Kinetic	$i_{\mathrm{Pd}}^{\mathrm{nucl}}-t^{3}$

## Data Availability

The data presented in this study are available on request from the corresponding author.
